# Honing in on phenotypes: comprehensive two-dimensional gas chromatography of herbivory-induced volatile emissions and novel opportunities for system-level analyses

**DOI:** 10.1093/aobpla/plt002

**Published:** 2013-01-21

**Authors:** Emmanuel Gaquerel, Ian T. Baldwin

**Affiliations:** Department of Molecular Ecology, Max-Planck-Institute for Chemical Ecology, Hans-Knöll-Str. 8, Jena 07745, Germany

**Keywords:** Herbivory, metabolomics, *Nicotiana attenuata*, plant volatiles, two-dimensional gas chromatography, untargeted analysis.

## Abstract

Plant volatile organic compound (VOC) production requires a complex network of biochemical pathways, which, although well mapped from a biochemical point of view, remains only partly understood with regard to its physiological and genetic regulation. Additionally, although analytical procedures for plant VOC measurement have become increasingly faster and more sensitive in recent years, pinpointing relevant shifts in VOC production from the thousands of molecular fragments that are generated by modern mass spectrometer instruments remains challenging. Here we discuss novel opportunities for system-wide analysis provided by the implementation of non-targeted data processing and multivariate statistics in VOC analysis. We illustrate the value of implementing non-targeted data processing with examples of recent findings from our group on the interactive control exerted by salivary components of a lepidopteran herbivore, *Manduca sexta*, on herbivory-induced VOC emissions in the wild tobacco *Nicotiana attenuata*. Finally, we briefly discuss the use of multi-platform data integration for probing the nature of metabolic and regulatory systems underlying VOC emissions.

## Abstract (precis)

Here we discuss opportunities for system-wide analysis of plant volatiles provided by the implementation of non-supervised data processing. We illustrate the value of such approaches by presenting recent findings on wild tobacco volatile emissions using two-dimensional gas chromatography.

## Introduction

Plants continuously emit large quantities of chemically highly diverse volatile organic compounds (VOCs). More than 1700 VOCs have, for instance, been detected in floral scents and many more are emitted from other tissues. These VOC blends mainly consist of products of the phenylpropanoid pathway, terpenoids and fatty acid (FA) and amino acid derivatives (reviewed in Baldwin 2010). Green leaf volatiles (GLVs), the best structurally characterized FA-based VOCs, are synthesized by all higher plants during mechanical damage. Their emission originates from the degradation of C_18_ FAs—linolenic and linoleic acids—when hydroperoxidated by a lipoxygenase (LOX) and cleaved into C_12_ and C_6_ units by a hydroperoxide lyase (HPL) (Halitschke *et al.* 2004; Matsui 2006). In contrast, terpenoids, which are produced by the mevalonic acid pathway in the cytosol or the 2-methylerythritol 4-phosphate/1-deoxy-xylulose 5-phosphate pathway located in the plastids, provide much of the structural diversity in plant VOC blends.

Volatile organic compounds are central to multiple aspects of a plant fitness in nature, notably by providing information to pollinators or competitors and by protecting plant tissues from biotic and abiotic stresses. The release of VOCs from vegetative organs following insect herbivore attack is a general property of plant species (reviewed in Turlings and Wäckers 2004) and has been the topic of numerous studies addressing their biosynthesis and release, as well as the eco-physiological functions of these emissions. Early studies and many others since then, conducted under optimized laboratory conditions (De Moraes *et al.* 1998; Turlings 1998 and for a review, Dicke 2000) or under field conditions (Kessler and Baldwin 2001), on this induced response have demonstrated that parasitoids and predators make effective use of herbivory-induced volatiles to locate their prey.

Tholl *et al.* (2006) recently reviewed currently available methods for plant volatile collection and analysis. As highlighted by the authors of the review, the number of analytes that can be detected in a single sample has increased considerably in recent years owing to impressive advances in the resolution and signal detection of modern gas chromatography coupled to mass spectrometers (GC–MS). However, our capacity to pinpoint relevant shifts in these large data-sets has not increased in tandem. This schism has been discussed in a review article by van Dam and Poppy (2008). The authors of this excellent review encourage the implementation of multivariate statistics and meta-analysis of plant VOCs, and notably its integration with insect behavioural data to increase the impact of modern instrumentation in understanding the ecological function of plant volatiles. Here, we describe the tools and resources already available for the untargeted post-processing and comparison of plant VOC profiles, and illustrate its value with examples of recent findings from our group on the interactive levels of control on herbivory-induced plant VOC (HIPV) emissions in *Nicotiana attenuata*. Finally, we briefly discuss novel avenues offered by multi-platform data integration.

## Implementing unsupervised data processing and statistical interpretation in VOC analysis

Plant VOC emissions are indicative of the physiological and metabolic status of the emitter. A better characterization of the plethora of homeostatic controls shaping plant VOC emissions is therefore essential for understanding their eco-physiological functions. To do so, one has first to discriminate between ecologically informative signals emitted by the plant and the surrounding noise. To succeed in this task, the implementation of rigorous data processing approaches and statistical analyses in VOC research is required. Here, we provide an overview of existing and readily implementable tools for the untargeted processing of GC–MS plant VOC data-sets. Such tools originate from the field of metabolomics—the omics approach interested in the comprehensive study of chemical fingerprints that specify cellular processes (Fiehn 2002)—for which numerous commercial and freeware programs have recently been developed. Commonly used programs perform raw file translation, pre-processing of mass chromatograms—including peak picking and deconvolution—and export peak tables suitable for statistical treatments. A non-exhaustive list of suitable programs is available at the web page of the Fiehn lab at UC Davis (http://fiehnlab.ucdavis.edu/staff/kind/Metabolomics/). Most of these programs can be readily applied to normal GC–MS data as they make use of the NetCDF file format (ASTM E2078-00, Standard Guide for Analytical Data Interchange Protocol for Mass Spectrometric Data), which can be produced from most, if not all, commercial file formats. File translation is usually performed by using platform-specific GC–MS operating software. Converters to a more recently developed exchangeable file format, such as mzXML, have been developed by both academic institutions and companies.

Typical post-processing pipelines for large MS data-sets proceed through multiple stages, with feature detection and alignment being the two most critical ones (Katajamaa and Oresic 2007). Feature detection, also referred to as ‘peak picking’, corresponds to the detection and extraction of the representations of measured ions from a GC–MS or a liquid chromatography coupled to mass spectrometer (LC–MS) raw signal. Alignment methods cluster extracted feature traces across different samples. This requires correcting for peak drifts to accurately determine the concentration of analytes in an automated fashion to produce peak tables suitable for cross-sample statistical comparisons. Many of the commercial or freeware programs developed in recent years attempt to bridge the gap existing between analytical chemistry and biology, and thereby to facilitate the rapid extraction of ecologically relevant signals from complex matrices. Among these programs are AMDIS, MetAlign (Lommen 2009), MZmine (and its update MZmine2; Katajamaa *et al.* 2006) and XCMS (Smith *et al.* 2006; and its online server, XCMS online, https://xcmsonline.scripps.edu/). The application of these programs to VOC profile analysis is discussed below.

AMDIS—an acronym that stands for Assisted Mass Deconvolution Identification Solution—is a computer program that extracts spectra for individual components in a GC–MS data file and identifies target compounds by matching these spectra against a reference library (Davies 1998). Deconvolution means grouping fragments corresponding to one mass spectrum, and thus a single compound, into clusters. This step in data processing is not only critical for database searches, but also reduces the redundancy of a peak table prior to multivariate statistics. MetAlign is another efficient data processing freeware for the pre-processing and comparison of full scan nominal or accurate mass LC–MS and GC–MS data. The new version of metAlign is available as a free download at www.metalign.nl. This program is capable of automatic file format conversions, accurate mass isolation, baseline corrections, peak picking and *m*/*z* feature artefact filtering, as well as alignment of up to 1000 data-sets. MetAlign has been effectively applied to the post-processing of GC–MS and LC–MS data-sets obtained from the quality control of fruits (Tikunov *et al.* 2005, 2010), fermentation studies (De Bok *et al.* 2011) and drinking water (Lommen *et al.* 2007). MZmine2 is another open-source project software dedicated to MS data processing. It is based on the original MZmine toolbox, described in Katajamaa *et al.* (2007), and proposes a user-friendly and flexible set of modules covering the entire work-flow pipeline, including visualization, peak picking, peak list processing, alignment and searching in a custom library or by connecting the PubChem Compound database as well as basic statistical tools. Unfortunately, MetAlign and MZmine2, like most peak picking programs, do not include options for mass spectrum deconvolution. Mass spectra must therefore be reconstructed from individually extracted molecular fragments. Tikunov *et al.* (2005) demonstrated, by investigating polymorphisms in fruit VOC emissions from tomato cultivars, that mass spectra reconstruction can be performed by confounding co-eluting molecular fragments sharing significant co-regulation across the data-set. In recent years, XCMS has become the most commonly used program to post-process LC–MS-based metabolomics data-sets (Smith *et al.* 2006; Tautenhahn *et al.* 2008). This bioconductor R package can be applied to GC–MS data analysis and the recently developed web-server (XCMS online) provides a set of parameters optimized for the processing of GC-based data. XCMS imports chromatographically or non-chromatographically separated and single-spectra and tandem mass spectral data from AIA/ANDI NetCDF, mzXML, mzData and mzML files. The XCMS analysis methodology involves peak picking/filtering and matching these peaks across samples for retention time correction. Differential *m/z* features—extracted ion chromatograms are automatically generated for a given number of them—or the complete *m/z* matrix are then exported as a tab-delimited file. XCMS can be used in combination with CAMERA, a bioconductor package that isolates compound-specific mass spectra according to the quality of the fit between extracted ion currents from co-eluting molecular fragments (Kuhl *et al.* 2011). BinBase represents another open-source program well suited for between-treatment comparative processing and automated identification of VOCs in complex mixtures (Skogerson *et al.* 2011).

All these MS processing programs export processed results into file formats suitable for most statistical packages. The application of multivariate statistics—a branch of exploratory statistics allowing the reduction of a large data-set on several mutually dependent variables—to the analysis of HIPV emissions has been comprehensively reviewed in van Dam and Poppy (2008). Noteworthy are the important advances made in recent years in the development of statistical web-servers, such as MetaboAnalyst (Xia 2011), that guide users through a step-by-step statistical analysis pipeline rich in a variety of menus, information hyperlinks and check boxes. The next paragraph demonstrates the value of mutualizing high chromatographic resolution and clustering statistics to access the complexity of the signalling events translating into HIPV emissions.

## GC × GC metabolomics reveals how insect saliva components reconfigure a plant's wound volatile response

**Fig. 1:**
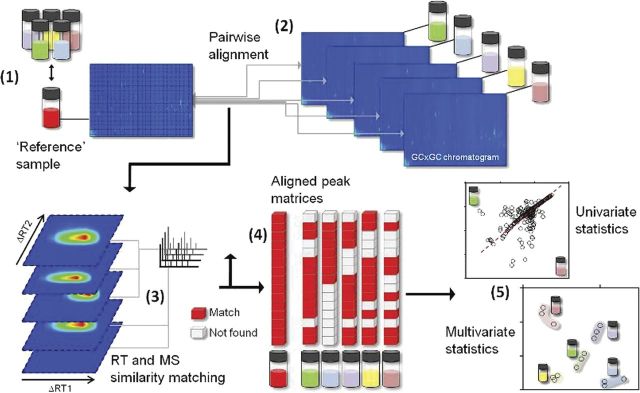
**An approach for non-targeted processing of GC × GC plant volatile maps.** Two-dimensional counter maps are generated after the analysis of volatile extracts by GC × GC–ToF-MS. Peak lists are produced using the peak picking and deconvolution tools of the instrument operating software and subsequently aligned separately (1) to a reference matrix issued from the analysis of an equimolar mixture of all samples (2). Peaks providing matches with metabolites from the reference matrix (4) are then retained for univariate and multivariate statistical analysis (5).

As described above, statistics can be queried to reveal how plants modulate their VOC emissions to identify and manipulate these emissions in an informed fashion. This particularly holds true for HIPV emissions as they are highly dynamic processes controlled by intricate networks of signalling and biosynthesis pathways. Herbivory-induced plant VOC emissions have only been comprehensively characterized in a few plant species (for an overview, see van Poecke and Dicke 2004; Van Dam and Poppy 2008; Baldwin 2010) including, for instance, maize (*Zea mays*), cotton (*Gossypium hirsutum*), lima bean (*Phaseolus lunatus*) and native tobacco (*N. attenuata*). Unbiased comparative analyses of HIPV blends may in some cases require exceptionally high resolution given the high structural diversity of its constituents and the range of concentrations at which they occur. Earlier studies in our group have exemplified the use of two-dimensional gas chromatography coupled to a time-of-flight mass spectrometer (GC × GC–ToF-MS), a technique which combines the advantages of high chromatographic resolution and sensitivity, for the analysis of HIPVs (Gaquerel *et al.* 2009). In order to advance our understanding of the mechanisms responsible for *N. attenuata*'s HIPV, we developed a work-flow suited for the comparative processing of GC × GC VOC maps (Gaquerel *et al.* 2009). This conceptual work-flow is depicted in Fig. 1. Raw chromatograms are first subjected to untargeted deconvolution (Fig. 1, step 1) using the commercial LECO ChromaToF software used to operate the instrument to extract analyte peaks from noise. Inconsistencies across sample specimens in the size of deconvoluted peak tables are, in a second step, corrected to generate a matrix format where rows represent an individual peak, columns an individual sample and the values are peak intensities obtained by integration of deconvoluted peaks (Fig. 1, steps 2–4). The comparison feature embedded in the LECO ChromaToF v 2.21 software can align different peak lists: this requires the comparison of each individual chromatogram to a unique reference matrix, ideally obtained by the analysis of an equimolar mixture of all sample specimens, in order to obtain output tables with the same numbers of peaks (Fig. 1, step 4). Newer versions of ChromaToF software allow more straightforward multi-parallel sample alignment and therefore do not require the analysis and generation of a reference matrix. This procedure provides, after mining with similar statistical analysis, relatively similar maps of differentially regulated VOCs as the reference matrix-based approach. We originally used this processing framework to parse HIPVs according to the eliciting effects of single components from *Manduca sexta* oral secretions (OS) (Gaquerel *et al.* 2009). In this study, we obtained, after comparative processing, a list of 400 analytes for cross-sample statistical analysis. Two-thirds of the extracted VOCs induced during simulated insect feeding had emissions that were dependent on the quality of *M. sexta* OS cues applied to the leaf surface. Clustering analyses revealed that the regulation of these VOC emissions occurred in modules. This previously published analysis confirmed the central role of *N*-linoleoylglutamine and *N*-linoleoylglutamate, two abundant fatty acid–amino acid conjugates (FACs) in OS, in either amplifying the wound response or inducing specific VOC emissions. But this work also underscored that additional, as yet unknown, factors, other than the influence of the alkaline pH of *M. sexta* OS (von Dahl *et al.* 2006), may shape these responses. Fatty acid–amino acid conjugates are herbivory-associated molecular patterns contained in the OS of many insect species and have been shown to activate a large array of herbivory-specific responses in plants (Bonaventure *et al.* 2011). In *N. attenuata*, FAC-inducible responses include kinase activation (Wu *et al.* 2007), amplification of oxylipin production (Kallenbach *et al.* 2010, 2011), major reconfigurations in transcript (Gilardoni *et al.* 2010), protein and metabolite pools (Halitschke *et al.* 2001), as well as translocation of recently fixed photo-assimilates to the roots (Schwachtje *et al.* 2006).

**Fig. 2:**
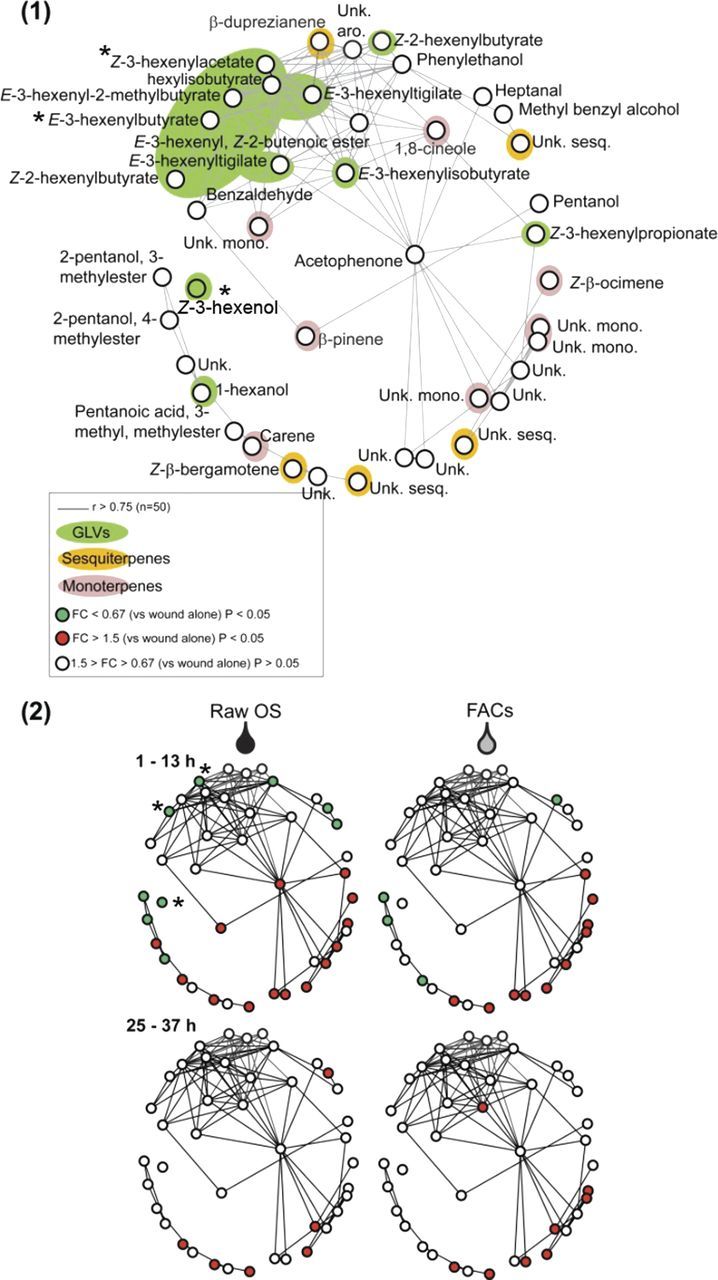
**Statistical analysis of non-targeted GC × GC–ToF-MS volatile profiles of *M. sexta* OS and FAC elicited leaves pinpoint insect-specific reconfigurations of the wound response.** Data used for the network analysis have been published in Gaquerel *et al.* (2009). *Nicotiana attenuata* leaf VOCs were collected 1–13 and 25–37 h after mechanical wounding and direct application of a buffer mimicking the pH of *M. sexta* OS (wounding alone), raw *M. sexta* OS diluted in distilled water or FACs, *M. sexta* OS elicitors activating, at physiological concentrations, many of the early responses observable during insect feeding. (1) Correlation-based network analysis using the overall sample set (*n* = 50 samples, issued from a compendium of six eliciting treatments, cf. Gaquerel *et al.* 2009) shows dependences in the regulation of VOC levels partly overlapping with large compound class definitions. Peaks obtained by non-targeted processing were reported at a signal-to-noise ratio of 10—since we estimated this value as the minimum required for integration and accurate identification during alignments with the NIST and homemade libraries—and matched across samples using the approach described in Fig. 1. Network visualization of correlation data was obtained with the Pajek software package (http://vlado.fmf.uni-lj.si/pub/networks/pajek/). Nodes represent identified metabolite belonging to the main VOC classes. The distance between two nodes is based on 1 − the absolute value of the Pearson correlation coefficient (*r*) with the Fruchtermann–Reingold three-dimensional algorithm. Nodes connected by an edge share a significant (*P* < 0.05) correlation coefficient *r* > 0.75. Significance levels for correlation coefficients were determined by following the number of metabolite pairs (*n*) using the equation *t* = *r* × (*n*−2)^0.5^/(1−*r*^2^)^0.5^ and the associated *P* value obtained from the *t* distribution table. (2) Mapping significant changes in VOCs released (compared to wounding alone) by *M. sexta* OS and FAC treatments onto the network highlights the dramatic control exerted by FACs over volatile terpenoid production and release. A VOC was considered significantly (unpaired *t*-test, *P* < 0.05) differentially emitted after treatment (*M. sexta* OS or FAC application) if its fold change (FC: ratio of means for treatment vs. control) was greater than 2 or lower than 0.5. Asterisks denote the position in the network of three VOCs reported in the main text.

To further visualize the modularity of *N. attenuata* HIPV emissions, we used a compendium of data measured after six different elicitations—data published in Gaquerel *et al.* (2009)—to construct a Pearson correlation-based network visualized using Pajek (Fig. 2, degree of freedom (d.o.f.) = 49). This network analysis highlights specific dependences in the regulation of VOC releases, which partly overlap with large compound class definition (Fig. 2A). As demonstrated earlier (Gaquerel *et al.* 2009), the comparative mapping of OS- and FAC-dependent regulation onto this network confirms that the early release of most GLVs as a result of FA oxidation and cleavage does not depend on FAC-based signalling. Hydrolytic enzymatic activities present in lepidopteran OS have long been argued to condition the type of VOCs being released during insect feeding (Mattiacci *et al.* 1995). In a recent study, Allmann and Baldwin (2010) demonstrated that an unknown heat-labile constituent of *M. sexta* OS catalyses the (*Z*)–(*E*) isomerization of GLVs produced directly in response to wounding of *N. attenuata* leaves, and that this phenomenon is not conditioned by the action of major phytohormonal transduction cascades. This shift in the GLV (*Z*)/(*E*) isomeric ratio increases the foraging efficiency of predators in nature (Allmann and Baldwin 2010). The reduction in (*Z*)-3-hexenylacetate, (*E*)-3-hexenylbutyrate and (*Z*)-3-hexenol levels as a consequence of *M. sexta* OS is also apparent in our network analysis (Fig. 2B, the localization of these three VOCs in the network is indicated by asterisks). The study performed by Allmann and Baldwin (2010) also illustrates the value of coupling unbiased VOC analysis with insect behavioural assays for the identification of ecologically relevant signals. The lower part of the network clusters together VOCs (monoterpenes, sesquiterpenes and unknowns) with emission levels amplified by FAC perception. Even though both are controlled by FAC signalling, emissions of herbivore-specific monoterpenes and sesquiterpenes probably result from distinct downstream regulatory cascades, as indicated by the different dynamics of the release of these two compound classes (Gaquerel *et al.* 2009).

**Fig. 3:**
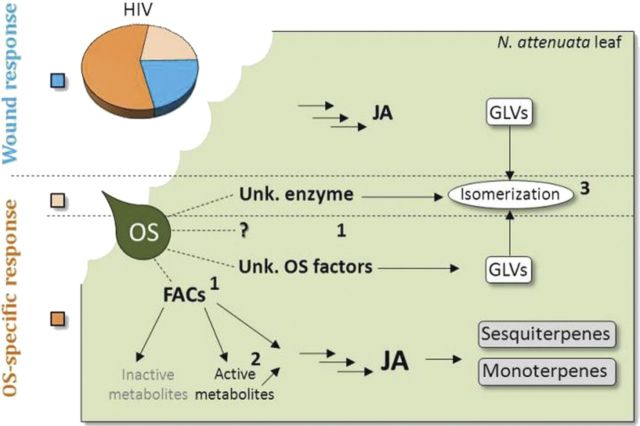
**Current knowledge of the modulations of the wound volatile response triggered by *M. sexta* OS cues in *N. attenuata*.** When *M. sexta* larvae chew on a leaf, OS contaminate wounds to elicit specific reconfigurations of the initial wound response (blue sector of the pie chart). Oral secretions activate the release of new VOCs and enhance or repress the initial wound response for other VOCs (light and dark orange of the pie chart). Jasmonic acid-based signalling plays a pivotal role in the activation of transcription-dependent terpenoid releases. Both the nature of the jasmonate signal(s) and the downstream elements controlling this response remain largely elusive. (1) Oral secretions  probably also contain yet to be identified additional factors qualitatively and quantitatively shaping VOC emissions. Fatty acid–amino acid conjugates are central to the enhancement of the wound response and for OS-specific releases of terpenoid compounds (Gaquerel *et al.* 2009). (2) Fatty acid–amino acid conjugates are additionally processed into active and inactive oxidized forms when penetrating wounded tissues. Among the active metabolites, one has been shown to have a moderating effect on monoterpene emissions (van Doorn *et al.* 2010). (3) An unknown enzyme present in *M. sexta* OS catalyses the (*Z*)–(*E*) isomerization of GLVs produced directly upon wounding of *N. attenuata* leaves. (*E*)-isomers are produced from plant-derived (*Z*)-isomers and increase the foraging efficiency of predators (Allmann and Baldwin 2010).

Figure 3 summarizes the current knowledge of our group on how *M. sexta* OS-dependent modulations of the wound VOC response take place in *N. attenuata* leaves. Parallel to the role of insect-derived enzymatic activities in framing a plant's VOC emissions, recent work has shown that insect-derived elicitors are subjected to oxidative processes by certain plant enzymes. Fatty acid–amino acid conjugates are quickly oxidized by a lipoxygenase isoform (LOX2) when penetrating insect-damaged leaf tissues (van Doorn *et al.* 2010). Interestingly, non-targeted comparative GC × GC–ToF-MS analysis demonstrated that 13-oxo-*N*-linoleoyl-glutamate, the major product of this oxidative metabolism, specifically enhances the emission of certain monoterpenes (van Doorn *et al.* 2010). The jasmonic acid (JA) pathway is the core signalling module regulating induced defences to insect herbivores in plants (Kessler *et al.* 2004), including the release of terpenoids (Kessler and Baldwin 2001). Multivariate analysis of VOC measurements carried out on transgenic lines silenced in the expression of downstream JA-responsive elements will probably shed new light on novel VOC branch-specific signalling and transcriptional regulatory mechanisms.

## Perspectives for metabolomics-assisted functional genomics of HIPV

Non-targeted analysis of plant VOC emissions offers new research opportunities in functional genomics, as we next discuss to highlight the value of integrating multiple omics approaches. Research in this direction might notably benefit from the development of multi-instrumental analytical platforms used to trace both the biogenesis of VOCs *in planta* and their emission into the air. For instance, Tikunov *et al.* (2010) combined GC–MS-based VOC measurements with non-targeted LC–ToF-MS profiling of tomato fruit cultivars. Intriguingly, the so-called ‘metabolic data fusion’ approach revealed that the reduced levels of emission, in certain tomato cultivars, of three phenylpropanoid volatiles translated from specific conversions of traditional hexose–pentoside precursors into glycoconjugates of higher complexity which were not cleaved upon fruit tissue disruption (Tikunov *et al.* 2010). The application of similar multi-instrumental approaches to HIPV emissions could theoretically pave the way for a better understanding of the mechanisms associated with VOC accumulation and release during plant–insect interactions.

Innovative research on the functional genomics of plant VOCs will undoubtedly benefit from convergences in high-throughput transcriptomics and metabolomics. A large body of recent literature reports (for a review, see Saito *et al.* 2008) has demonstrated the value of integrating these two omics approaches by means of data-mining informatics. This strategy represents an excellent tool for the prediction and identification of novel genes involved in complicated metabolic pathways. Originally applied to *Arabidopsis* stress responses, detailed informatics mining of gene expression profiling data and targeted metabolite measurements induced by deficiency of sulfur and nitrogen led to the identification of previously unknown molecular players involved in glucosinolate biosynthesis (Hirai *et al.* 2005). The approach used in this study and many others that have followed is largely based on the ‘guilt-by-association’ principle, which assumes that a shared regulatory control is exerted over genes involved in a core biological process. The application of this strategy for gene function prediction has been excellently reviewed by Saito *et al.* (2008). Gene-to-metabolite integrative analyses have been applied in a few cases for VOC biosynthetic gene annotation, notably for the genes controlling developmentally regulated VOC releases by strawberry (*Fragaria* sp.; Aharoni *et al.* 2000) and spider mite-induced responses in cucumber (*Cucumis sativus*; Mercke *et al.* 2004) or for those responsible for polymorphisms in VOC emissions detected in different accessions and introgression lines of wild and cultivated tomato (*Lycopersicon hirsutum* and *Solanum lycopersicum*; Tikunov *et al.* 2005, 2010). The latter study also underlines the power of exploiting natural variation in identifying the genes responsible for VOC production. Unfortunately, only a few such studies have addressed natural variations in the HIPV emissions from non-domesticated species, for example in *Datura wrightii* (Hare *et al.* 2007) or *N. attenuata* (Schuman *et al.* 2009). We have identified large variations, probably genetically determined, in the release of most HIPV in a native population of *N. attenuata* plants growing in Utah (USA) (Schuman *et al.* 2009). These quantitative variations in HIPV emissions were only partially associated with expression-level polymorphisms detected for JA biosynthetic and perception genes, suggesting that significant polymorphisms in VOC biosynthetic genes accounted for the VOC genetic diversity of *N. attenuata* native populations.

## Conclusions and forward look

In conclusion, the implementation of unsupervised processing and statistics has the power to increase the impact of modern instrumentation in understanding the chemical ecology of plant VOCs. Succeeding in this task implies rigorous data processing and statistical mining following standards established in the last decade for microarray analysis. Here, we provided a rapid overview on open access and commercial programs to perform these analyses. The choice of one of these programs depends on the type of data produced, on users' computational experience but also on later processing steps (including statistical analysis) to be conducted with the output matrix. In this respect, the R platform offers an extremely large and versatile statistical environment for mining analyte matrices produced in a first step by packages such as XCMS. Like most automated data processing procedures, peak picking and alignment steps are prone to false-positive and false-negative assignments, materialized, for instance, by non-extracted true analyte peaks, over-representation of noise-associated features and non-correctly aligned mass features. The selection of the best algorithm, but also of the best associated parameters, is therefore not an easy task since different settings strongly affect peak detection/alignment performance. Thus, quality control tests—some of these procedures being documented as part of software/package manuals—as well as manual inspection of the output data are important steps to understand and optimize data-processing and to conduct rigorous statistical analysis.

Embedding the processing and statistical analysis results within the context of existing biological knowledge represent another main challenge of metabolomics studies. Global metabolic profiles or fingerprints can be a very powerful means of comparing biological samples; however, metabolite identity and data integration (different metabolomics platforms, metabolomics–genomics data) remain indispensable for providing mechanistic insight into a biological phenomenon. In this direction, integration of metabolomics data obtained from different analytical platforms is becoming crucial to get a broad overview on biochemical shifts associated with HIPV emissions, for instance by assessing stress- or developmentally-regulated modulations of  non-volatile precursor pools using LC-based analytics (Tikunov *et al.* 2010). Recent work on plant–insect interaction has also shown the importance of conducting real-time measurements of low-molecular-weight compounds such as ethylene (laser-based infrared photo-acoustic detection) (Von Dahl *et al.* 2007) or methanol (proton transfer reaction–mass spectrometry) (Von Dahl *et al.* 2006; Körner *et al.* 2009) in parallel with the assessment of traditional herbivory-induced metabolic pathways (including HIPV) to understand the complexity of plant defence against insects. For all these reasons, parallel genetic analyses of physiological, transcriptional and analytical measures of HIPV emissions are needed to enhance our ability to pinpoint some of the causal genes in plant signalling and metabolic cascades shaping HIPV bouquets.

## Sources of funding

This research was supported by the Max Planck Society.

## Contributions by the authors

E.G. and I.T.B. wrote the manuscript. Both authors have seen and agreed to the final submitted manuscript.

## Conflicts of interest statement

None declared.

## References

[PLT002C1] Aharoni A, Keizer LCP, Bouwmeester HJ, Sun Z, Alvarez-Huerta M, Verhoeven HA, Blaas J (2000). Identification of the SAAT gene involved in strawberry flavor biogenesis by use of DNA microarrays. The Plant Cell.

[PLT002C2] Allmann S, Baldwin IT (2010). Insects betray themselves in nature to predators by rapid isomerization of green leaf volatiles. Science.

[PLT002C4] Baldwin IT (2010). Plant volatiles. Current Biology.

[PLT002C6] Bonaventure G, VanDoorn A, Baldwin IT (2011). Herbivore-associated elicitors: FAC signaling and metabolism. Trends in Plant Science.

[PLT002C7] Davies AN (1998). The new Automated Mass Spectrometry Deconvolution and Identification System (AMDIS). Spectroscopy Europe.

[PLT002C8] De Bok FAM, Janssen PWM, Bayjanov JR, Sieuwerts S, Lommen A, Van Hylckama Vlieg JET, Molenaar D (2011). Volatile compound fingerprinting of mixed-culture fermentations. Applied and Environmental Microbiology.

[PLT002C9] De Moraes CM, Lewis WJ, Pare PW, Alborn HT, Tumlinson JH (1998). Herbivore-infested plants selectively attract parasitoids. Nature.

[PLT002C10] Dicke M (2000). Chemical ecology of host-plant selection by herbivorous arthropods: a multitrophic perspective. Biochemical Systematics and Ecology.

[PLT002C11] Fiehn O (2002). Metabolomics—the link between genotypes and phenotypes. Plant Molecular Biology.

[PLT002C12] Gaquerel E, Weinhold A, Baldwin IT (2009). Molecular interactions between the specialist herbivore *Manduca sexta* (Lepidoptera, Sphigidae) and its natural host *Nicotiana attenuata*. VIII. An unbiased GC×GC-ToFMS analysis of the plant's elicited volatile emissions. Plant Physiology.

[PLT002C13] Gilardoni PA, Schuck S, Jüngling R, Rotter B, Baldwin IT, Bonaventure G (2010). SuperSAGE analysis of the *Nicotiana attenuata* transcriptome after fatty acid–amino acid elicitation (FAC): identification of early mediators of insect responses. BMC Plant Biology.

[PLT002C14] Halitschke R, Schittko U, Pohnert G, Boland W, Baldwin IT (2001). Molecular interactions between the specialist herbivore *Manduca sexta* (Lepidoptera, Sphingidae) and its natural host *Nicotiana attenuata*. III. Fatty acid–amino acid conjugates in herbivore oral secretions are necessary and sufficient for herbivore-specific plant responses. Plant Physiology.

[PLT002C15] Halitschke R, Ziegler J, Keinanen M, Baldwin IT (2004). Silencing of hydroperoxide lyase and allene oxide synthase reveals substrate and defense signaling crosstalk in *Nicotiana attenuata*. The Plant Journal.

[PLT002C16] Hare DJ (2007). Variation in herbivore and methyl jasmonate-induced volatiles among genetic lines of *Datura wrightii*. Journal of Chemical Ecology.

[PLT002C17] Hirai MY, Klein M, Fujikawa Y, Yano M, Goodenowe DB, Yamazaki Y, Kanaya Y, Saito K (2005). Elucidation of gene-to-gene and metabolite-to-gene networks in *Arabidopsis* by integration of metabolomics and transcriptomics. The Journal of Biological Chemistry.

[PLT002C18] Kallenbach M, Alagna F, Baldwin IT, Bonaventure G (2010). *Nicotiana attenuata* SIPK, WIPK, NPR1, and fatty acid–amino acid conjugates participate in the induction of jasmonic acid biosynthesis by affecting early enzymatic steps in the pathway. Plant Physiology.

[PLT002C19] Kallenbach M, Gilardoni PA, Allmann S, Baldwin IT, Bonaventure G (2011). C_12_ derivatives of the hydroperoxide lyase pathway are produced by product recycling through lipoxygenase-2 in *Nicotiana attenuata* leaves. New Phytologist.

[PLT002C20] Katajamaa M, Oresic M (2007). Data processing for mass spectrometry-based metabolomics. Journal of Chromatography A.

[PLT002C21] Katajamaa M, Miettinen J, Oresic M (2006). MZmine: toolbox for processing and visualization of mass spectrometry based molecular profile data. Bioinformatics.

[PLT002C22] Kessler A, Baldwin IT (2001). Defensive function of herbivore-induced plant volatile emissions in nature. Science.

[PLT002C23] Kessler A, Halitschke R, Baldwin IT (2004). Silencing the jasmonate cascade: induced plant defenses and insect populations. Science.

[PLT002C24] Körner E, von Dahl CC, Bonaventure G, Baldwin IT (2009). Pectin methylesterase NaPME1 contributes to the emission of methanol during insect herbivory and to the elicitation of defence responses in *Nicotiana attenuata*. Journal of Experimental Botany.

[PLT002C25] Kuhl C, Tautenhahn R, Böttcher C, Larson TR, Neumann S (2013). CAMERA: an integrated strategy for compound spectra extraction and annotation of LC/MS data sets. Analytical Chemistry.

[PLT002C26] Lommen A (2009). MetAlign: interface-driven, versatile metabolomics tool for hyphenated full-scan mass spectrometry data preprocessing. Analytical Chemistry.

[PLT002C27] Lommen A, Van Der Weg G, Van Engelen MC, Bor G, Hoogenboom LAP, Nielen MWG (2007). An untargeted metabolomics approach to contaminant analysis: pinpointing potential unknown compounds. Analytica Chimica Acta.

[PLT002C28] Matsui K (2006). Green leaf volatiles: hydroperoxide lyase pathway of oxylipin metabolism. Current Opinion in Plant Biology.

[PLT002C29] Mattiacci L, Dicke M, Posthumus MA (1995). beta-Glucosidase: an elicitor of herbivore-induced plant odor that attracts host-searching parasitic wasps. Proceedings of the National Academy of Sciences of the USA.

[PLT002C30] Mercke P, Kappers IF, Verstappen FWA, Vorst O, Dicke M, Bouwmeester HJ (2004). Combined transcript and metabolite analysis reveals genes involved in spider mite induced volatile formation in cucumber plants. Plant Physiology.

[PLT002C31] Saito K, Hirai MY, Yonekura-Sakakibara K (2008). Decoding genes with coexpression networks and metabolomics—‘majority report by precogs. Trends in Plant Science.

[PLT002C32] Schuman MC, Heinzel N, Gaquerel E, Svatos A, Baldwin IT (2009). Polymorphism in jasmonate signaling partially accounts for the variety of volatiles produced by *Nicotiana attenuata* plants in a native population. New Phytologist.

[PLT002C33] Schwachtje J, Minchin PEH, Jahnke S, Dongen V, Schittko U, Baldwin IT (2006). SNF1-related kinases allow plants to tolerate herbivory by allocating carbon to roots. Proceedings of the National Academy of Sciences of the USA.

[PLT002C34] Skogerson K, Wohlgemuth G, Barupal DK, Fiehn O (2011). The volatile compound BinBase mass spectral database. BMC Bioinformatics.

[PLT002C35] Smith CA, Want EJ, O'Maille G, Abagyan G, Siuzdak G (2006). XCMS: processing mass spectrometry data for metabolite profiling using nonlinear peak alignment, matching, and identification. Analytical Chemistry.

[PLT002C36] Tautenhahn R, Böttcher C, Neumann S (2008). Highly sensitive feature detection for high resolution LC/MS. BMC Bioinformatics.

[PLT002C37] Tholl D, Boland W, Hansel A, Loreto F, Röse USR, Schnitzler JP (2006). Practical approaches to plant volatile analysis. The Plant Journal.

[PLT002C38] Tikunov Y, Lommen A, De Vos CHR, Verhoeven HA, Bino RJ, Hall RD, Bovy AG (2005). A novel approach for nontargeted data analysis for metabolomics. Large-scale profiling of tomato fruit volatiles. Plant Physiology.

[PLT002C39] Tikunov YM, De Vos RCH, González Paramás AXM, Hall RD, Bovy AG (2010). A role for differential glycoconjugation in the emission of phenylpropanoid volatiles from tomato fruit discovered using a metabolic data fusion approach. Plant Physiology.

[PLT002C40] Turlings TCJ (1998). The induction of volatile emissions in maize by three herbivore species with different feeding habits: possible consequences for their natural enemies. Biological Control.

[PLT002C42] Van Dam NM, Poppy GM (2008). Why plant volatile analysis needs bioinformatics—detecting signal from noise in increasingly complex profiles. Plant Biology.

[PLT002C43] Van Doorn A, Kallenbach M, Borquez AA, Baldwin IT, Bonaventure G (2010). Rapid modification of the insect elicitor *N*-linolenoyl-glutamate via a lipoxygenase-mediated mechanism on *Nicotiana attenuata* leaves. BMC Plant Biology.

[PLT002C44] Van Poecke RMP, Dicke M (2004). Indirect defence of plants against herbivores: using *Arabidopsis thaliana* as a model plant. Plant Biology.

[PLT002C45] Von Dahl CC, Hävecker M, Schlögl R, Baldwin IT (2006). Caterpillar-elicited methanol emission: a new signal in plant–herbivore interactions?. The Plant Journal.

[PLT002C46] Von Dahl CC, Winz R, Halitschke R, Kühnemann F, Gase K, Baldwin IT (2007). Tuning the herbivore-induced ethylene burst: the role of transcript accumulation and ethylene perception in *Nicotiana attenuata*. The Plant Journal.

[PLT002C47] Wu J, Hettenhausen C, Meldau S, Baldwin IT (2007). Herbivory rapidly activates MAPK signaling in attacked and unattacked leaf regions but not between leaves of *Nicotiana attenuata*. The Plant Cell.

[PLT002C48] Xia J, Wishart DS (2011). Web-based inference of biological patterns, functions and pathways from metabolomic data using MetaboAnalyst. Nature Protocols.

